# Novel inflammatory mediator profile observed during pediatric heart surgery with cardiopulmonary bypass and continuous ultrafiltration

**DOI:** 10.1186/s12967-023-04255-8

**Published:** 2023-07-05

**Authors:** Joel Bierer, Roger Stanzel, Mark Henderson, Suvro Sett, John Sapp, Pantelis Andreou, Jean S. Marshall, David Horne

**Affiliations:** 1 Division of Cardiac Surgery, Dalhousie University, Halifax, Canada; 2 Department of Clinical Perfusion, Nova Scotia Health Authority, Halifax, Canada; 3 Division of Cardiology, Dalhousie University, Halifax, Canada; 4 Department of Community Health & Epidemiology, Dalhousie University, Halifax, Canada; 5 Department of Microbiology & Immunology, Dalhousie University, Halifax, Canada

**Keywords:** Pediatric cardiac surgery, Congenital heart disease, Cardiopulmonary bypass, Complement, Inflammation, Ultrafiltration

## Abstract

**Background:**

Cardiopulmonary bypass (CPB) is associated with systemic inflammation, featuring increased levels of circulating pro-inflammatory cytokines. Intra-operative ultrafiltration extracts fluid and inflammatory factors potentially dampening inflammation-related organ dysfunction and enhancing post-operative recovery. This study aimed to define the impact of continuous subzero-balance ultrafiltration (SBUF) on circulating levels of major inflammatory mediators.

**Methods:**

Twenty pediatric patients undergoing cardiac surgery, CPB and SBUF were prospectively enrolled. Blood samples were collected prior to CPB initiation (Pre-CPB Plasma) and immediately before weaning off CPB (End-CPB Plasma). Ultrafiltrate effluent samples were also collected at the End-CPB time-point (End-CPB Effluent). The concentrations of thirty-nine inflammatory factors were assessed and sieving coefficients were calculated.

**Results:**

A profound increase in inflammatory cytokines and activated complement products were noted in plasma following CBP. Twenty-two inflammatory mediators were detected in the ultrafiltrate effluent. Novel mediators removed by ultrafiltration included cytokines IL1-Ra, IL-2, IL-12, IL-17A, IL-33, TRAIL, GM-CSF, ET-1, and the chemokines CCL2, CCL3, CCL4, CXCL1, CXCL2 and CXCL10. Mediator extraction by SBUF was significantly associated with molecular mass < 66 kDa (Chi^2^ statistic = 18.8, Chi^2^ with Yates’ correction = 16.0, p < 0.0001). There was a moderate negative linear correlation between molecular mass and sieving coefficient (Spearman R = − 0.45 and p = 0.02). Notably, the anti-inflammatory cytokine IL-10 was not efficiently extracted by SBUF.

**Conclusions:**

CPB is associated with a burden of circulating inflammatory mediators, and SBUF selectively extracts twenty of these pro-inflammatory factors while preserving the key anti-inflammatory regulator IL-10. Ultrafiltration could potentially function as an immunomodulatory therapy during pediatric cardiac surgery.

*Trial registration* ClinicalTrials.gov, NCT05154864. Registered retrospectively on December 13, 2021. https://clinicaltrials.gov/ct2/show/record/NCT05154864.

**Supplementary Information:**

The online version contains supplementary material available at 10.1186/s12967-023-04255-8.

## Introduction

Cardiopulmonary bypass (CPB) can be associated with a systemic inflammatory response in infants and children undergoing cardiac surgery [1, 2]. This is thought to be initiated by the alternative complement pathway reacting to the non-endothelialized CPB circuit and is enhanced by both coagulation cascade dysregulation and myocardial ischemia–reperfusion injuries during aortic cross-clamp time [1–3]. Complement activation rapidly produces C3a and C5a, which are potent anaphylatoxins, neutrophil activators and can stimulate the release of several cytokines and chemokines [4, 5]. Specifically, IL-6, a key inducer of acute phase reactants, and the neutrophil chemoattractant CXCL8 (IL-8) are well known to be released during CPB [6–8]. Collectively, CPB-associated inflammation can culminate in vasoplegia, capillary leak syndrome, distributive shock and organ dysfunction, which is prohibitive to a timely post-operative recovery [1, 9].

Pharmacologic and perfusion strategies have been developed in an effort to dampen CPB-associated inflammation. The impact of prophylactic glucocorticoids has been examined in randomized clinical studies but have yielded conflicting results, without consistent benefit for children undergoing cardiac surgery [2, 10]. Importantly, a recent landmark trial randomized 1263 infants to receive methylprednisolone or placebo failed to show superiority in the composite outcome of death, heart transplantation or 13 major post-operative outcomes [11]. In an attempt to modify complement responses, monoclonal antibody blockade of C5 has been investigated during adult coronary bypass surgery and CPB but showed no benefit in the primary composite outcome of death, new myocardial infarction, left ventricular dysfunction or new central nervous system deficit [12].

Intra-operative ultrafiltration has been used since the late 1980s to ameliorate the toxic response to CPB. Essentially, a portion of blood flow is shunted to a hemoconcentrator within the CPB circuit that is intended to remove fluid and small molecules, such as activated complement factors and inflammatory cytokines, with molecular mass up to 66 kDa [1, 13]. There are many ultrafiltration protocols, for the current study we used a continuous form of ultrafiltration called subzero-balance ultrafiltration (SBUF) which provides an opportunity to effectively assess changes in mediator levels in plasma and ultrafiltrate during the entire CPB exposure [1, 13].

In the published literature, ultrafiltration is often stated to remove inflammatory mediators; however, there is a lack of quantitative data regarding which inflammatory mediators are extracted [1, 14]. To date, C3a, C5a, terminal complement complex (TCC), IL-6, IL-8, IL-10, tumor necrosis factor (TNF), and neutrophil elastase have been directly measured in the ultrafiltrate effluent (Table 1) [15–22]. However, multiple other mediators participate in or regulate the innate immune response relevant to CPB-associated inflammation. The purpose of this study is to further contribute to the understanding of continuous forms of ultrafiltration as a potential immunomodulatory therapy and identify opportunities to advance the effectiveness of this technique.

**Table 1:** Known inflammatory mediators detected in ultrafiltration effluent

Publication	Year	Method	Mediators
Andreasson et al*.* [15]	1993	MUF	C3a and C5a
Saatvedt et al*.* [16]	1996	MUF	C3a and TNF
Saatvedt et al*.* [17]	1996	MUF	IL-8
Wang et al*.* [18]	1996	CUF and MUF	Elastase, TNF, IL-6 and IL-8
Wang et al*.* [19]	1998	MUF	TNF
Watanabe et al*.* [20]	1998	CUF	IL-6 and IL-8
Berdat et al*.* [21]	2004	CUF and MUF	TCC, IL-6, IL-10 and TNF
Lang et al*.* [22]	2014	MUF	IL-8 and IL-10

## Methods

Written informed consent was obtained from substitute decision-makers for all participants under a protocol approved by the IWK Health Centre Research Ethics Board (#1024869). The patients included in this analysis completed the study protocol between August 2020 and December 2020. This study is registered as NCT05154864 on ClinicalTrials.gov.

### Study design

Patients weighing < 30 kg undergoing cardiac surgery with CPB were prospectively enrolled pre-operatively and followed until post-operative discharge from the pediatric intensive care unit (PICU). All patients underwent their scheduled cardiac procedure with standard CPB, SBUF-SMUF and routine institutional post-operative medical management. Baseline clinical information was recorded from the medical record. Intraoperative data including CPB-time, cross-clamp time, type of CPB prime, transfusions, ultrafiltration volumes and fluid balance was collected from the perfusion record. Arterial blood (1 mL) was drawn post-sternotomy but prior to CPB initiation (Pre-CPB Plasma), and a second 1 mL of arterial blood was drawn at the end of CPB and SBUF (End-CPB Plasma) but prior to SMUF. Ultrafiltrate effluent (End-CPB Effluent) was collected simultaneously with the End-CPB Plasma as a paired sample.

### CPB and SBUF-SMUF technique

A comprehensive technical overview of our pediatric CPB with SBUF-SMUF has been reported [13]. A *Liva Nova S5™* CPB System (48-40-00, London, UK), *Terumo FX05* or *FX15* oxygenators (1CX*FX05RE/1CX*FX15E, Tokyo, Japan) and *Terumo Capiox*^*®*^ Hemoconcentrator HCO5 (1CX*HC05S, Tokyo, Japan) were used. Per the manufacturer, this ultrafiltration device has a sieving Coefficient of 0.2% for albumin, which has a molecular weight of 66 kDa. Sanguineous CPB prime was used for patients < 10 kg, while a crystalloid prime was used for those > 10 kgs. Buffered ultrafiltration of the CPB circuit prime (BUF) was used to normalize metabolic abnormalities prior to CPB initiation [23]. CPB was initiated after systemic heparinization achieved activated clotting time (ACT) > 480 s. Once full-flow was reached, SBUF was initiated for the remainder of CPB in a post-pump pre-oxygenator veno-venous fashion; 30 ml/kg/hr of effluent was removed while 25 ml/kg/hr of a physiologic solution was infused to target a net balance of -5 ml/kg/hr [13]. Cardioplegia and surgical field irrigation volumes were also removed via the hemoconcentrator. SBUF was paused during deep hypothermic circulatory arrest (DHCA). Just prior to weaning the patient from CPB, SBUF was deactivated. Immediately after separation from bypass, SMUF was initiated in a veno-arterial fashion with an endpoint target of venous reservoir depletion or reaching goal hematocrit of 40 [13]. For both SBUF and SMUF, 5% of calculated cardiac output was shunted to the hemoconcentrator. There was routine point-of-care blood gas and ACT monitoring throughout each patient’s surgery.

### Immunoanalysis

Arterial blood samples were collected in EDTA tubes, while effluent samples were collected in uncoated syringes. Effluent samples were divided into aliquots, flash-frozen in liquid nitrogen and stored at -80˚C. Arterial blood samples were centrifuged for 10 min (0.5 × gravity), and the resulting plasma was extracted. The plasma underwent a second centrifugation for 20 min (2.5 × gravity) to yield a platelet-free plasma which was aliquoted, flash-frozen in liquid nitrogen and stored at − 80 °C.

*Luminex* immunoanalysis of a panel of relevant mediators was completed with a *Bio-Rad Bio-Plex*^*®*^* 200* System (Hercules, United States). Thirty-nine pre-specified human inflammatory factors were analyzed using multiple analysis kits including: *ThermoFisher* C3a Simplex Kit (EPX010-12282-901, Waltham, United States), *Millipore Sigma* Human Complement Magnetic Beat Panel 1 (HCMP1MAG-19K-05, Burlington, United States), *Millipore Sigma* Human Complement Magnetic Beat Panel 2 (HCMP2MAG-19K-06, Burlington, United States), *BioTechne R&D Systems* Human XL Cytokine Luminex Performance Panel (FCSTM18-21, Minneapolis, United States), *BioTechne R&D Systems* Human Magnetic Luminex Assay (LXSAHM-05, Minneapolis, United States) and *BioTechne R&D Systems* Human Magnetic Luminex Assay (LXSAHM-01, Minneapolis, United States). All assays were completed pursuant to the manufacturer’s instructions. *Bio-Rad* Bio-Plex^®^ Manager™ Software 6.2 (Hercules, United States) was used to complete the data acquisition and used Logistic—5PL regression for all analytes.

### Statistical analysis

All data is presented as the median and interquartile range (IQR) because variables showed non-normal distributions. Analyte concentrations between the Pre-CPB Plasma sample and End-CPB Plasma sample were directly compared by Wilcoxon signed-rank test. The analyte’s sieving coefficient was calculated by dividing the End-CPB Effluent analyte concentration by the End-CPB Plasma analyte concentration and is expressed as a percentage. The association between ultrafiltration extraction (extracted vs. not extracted) and molecular mass (< 66 kDa vs. > 66 kDa) was assessed by the Chi-Squared test. Spearman’s rank correlation coefficient assessed the linear association between molecular mass and logarithmic transformation of sieving coefficient for those mediators less than 66 kDa. For the correlation analysis, mediators with sieving coefficient = 0 were assigned a log sieving coefficient of − 4. Inclusion of this subset of mediators, was important to not bias the results of the correlation analysis. A post-hoc analysis was conducted to compare the impact of intra-operative glucocorticoid use on mediator End-CPB plasma concentrations and sieving coefficients. The demographics and mediator sieving coefficients of glucocorticoid and non-glucocorticoid groups were compared by Wilcoxon rank-sum test and Chi-squared test. Statistical significance was defined as p < 0.05.

## Results

### Patient population and clinical data

During the study period, 20 pediatric patients were enrolled and completed the protocol. Baseline characteristics of the group are summarized in Table 2. The majority of patients were male (65%), less than one-year-old (70%) and had a variety of cardiac pathologies with the Society of Thoracic Surgeons-European Association for Cardio-Thoracic Surgery (STAT) risk scores between 1 and 4. Diagnoses included ventricular septal defect (VSD), partial and complete Atrioventricular Septal Defect (AVSD), Tetralogy of Fallot (TOF), d-Transposition of the Great Arteries (d-TGA), Truncus Arteriosus, Aorto-Pulmonary Window, both infra- and supra-cardiac Total Anomalous Pulmonary Venous Return (TAPVR) as well as single ventricle patients undergoing Glenn or Fontan operations. Half of the patients received steroids as part of their care on the day of operation with a median prednisone equivalent of 14.5 mg/kg. Operative and perfusion data are summarized in Table 3. There were no intra-operative perfusion- or ultrafiltration-related complications.

**Table 2:** Patient demographics (n = 20)

	No. (%), Median [IQR]
Sex	
Male	13 (65%)
Female	7 (35%)
Age (months)	4.0 [0.2–12.0]
Neonate (< 30 days)	7 (35%)
Infant (30 days–1 year)	7 (35%)
Child (> 1 year)	6 (30%)
Single ventricle	4 (20%)
STAT score	
1	4 (20%)
2	6 (30%)
3	2 (10%)
4	8 (40%)
Weight (kg)	5.2 [3.4–9.1]
≤ 10 kg	16 (80%)
> 10 kg	4 (20%)
Body surface area (m^2^)	0.30 [0.22–0.41]

**Table 3:** Intra-operative data (n = 20)

	No. (%), Median [IQR]
Steroid administration (count, prednisone equivalent mg/kg)	10 (50%), 14.5 [11.3–20.9]
CPB time (count, minutes)	20 (100%), 193 [158–272]
Cross-Clamp time (count, minutes)	18 (90%), 92 [73–124]
DHCA (count, minutes)	5 (25%), 29 [27–60]
Lowest temperature (°C)	28.0 [24.5–28.0]
SBUF-SMUF	
SBUF	20 (100%)
SMUF	19 (95%)
SBUF ultrafiltrate volume (ml/kg)	160 [104–228]
Urine output on CPB (ml/kg)	31 [18–48]
CPB volume balance (ml/kg)^a^	− 9 [− 3 to − 27]

*CPB* cardiopulmonary bypass; *DHCA* deep hypothermic circulatory arrest; *SBUF* subzero balance ultrafiltration; *SMUF* simple modified ultrafiltration

^a^CPB Volume Balance includes both SBUF and SMUF

### Complement pathway derived mediators

**Fig. 1:**
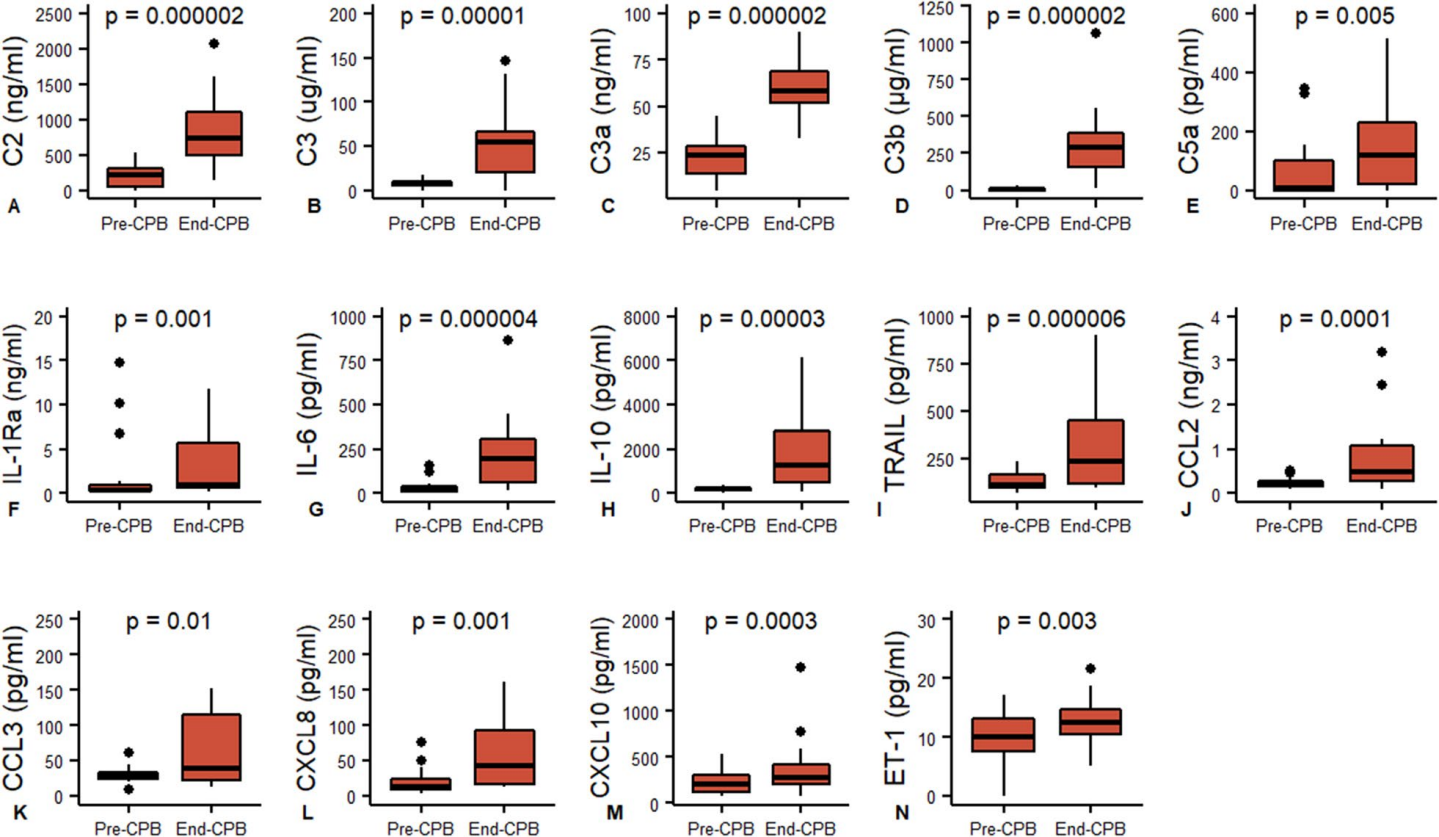
Inflammatory mediators that increase throughout CPB with SBUF. Paired comparison by Wilcoxon signed-rank test. C3a, C5a, IL-1Ra, IL-6, IL-10 CXCL8 (IL-8), CCL2, CCL3, CXCL10, TRAIL and ET1 were extracted by SBUF while C2, C3 and C3b were not

**Fig. 2:**
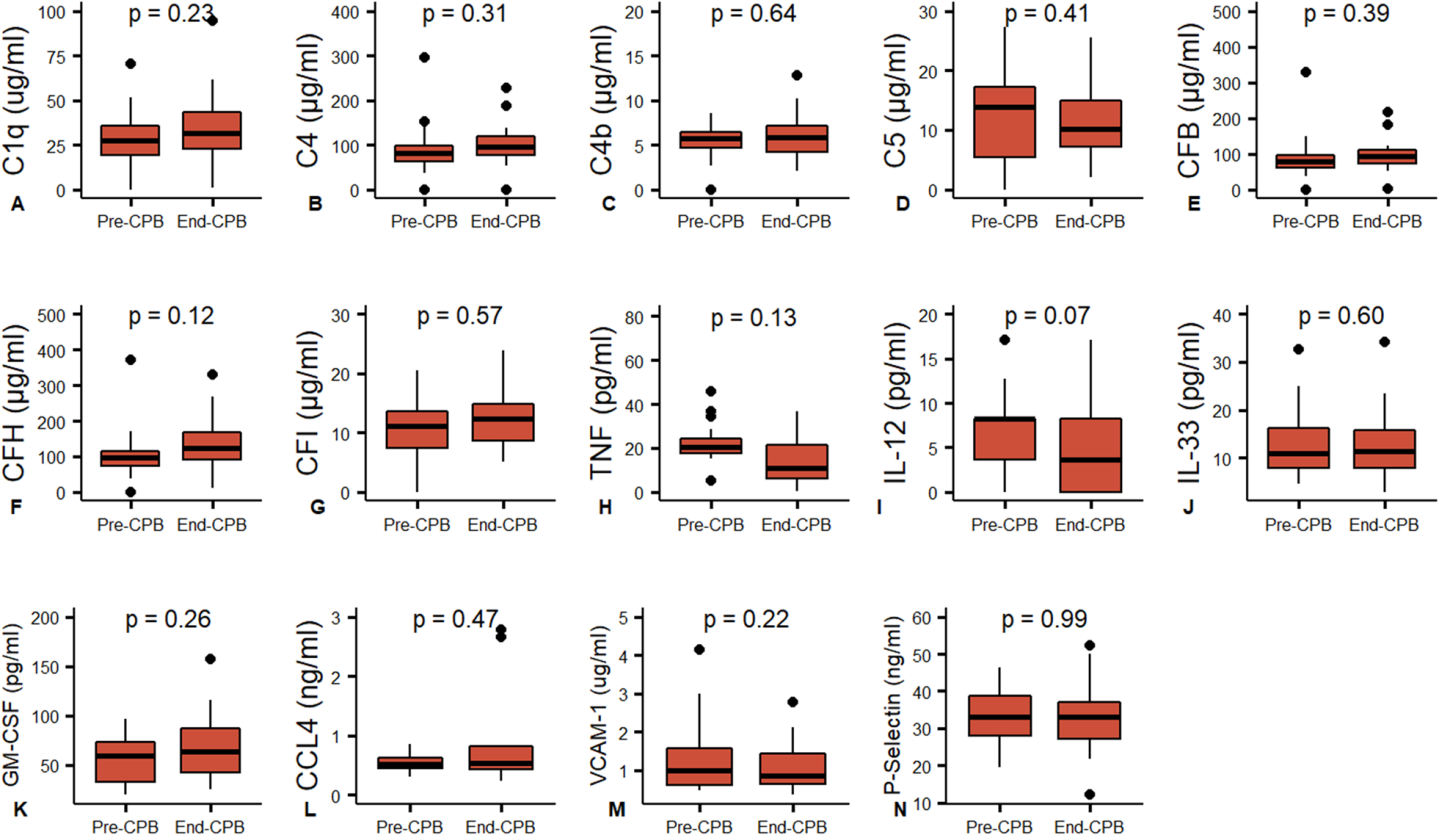
Inflammatory mediators that are constant throughout CPB with SBUF. Paired comparison by Wilcoxon signed-rank test. TNF, IL-12, IL-33, GM-CSF and CCL4 were extracted by SBUF while C1q, C4, C4b, C5, CFB, CFH, CFI, P-Selectin and VCAM-1 were not

**Fig. 3:**
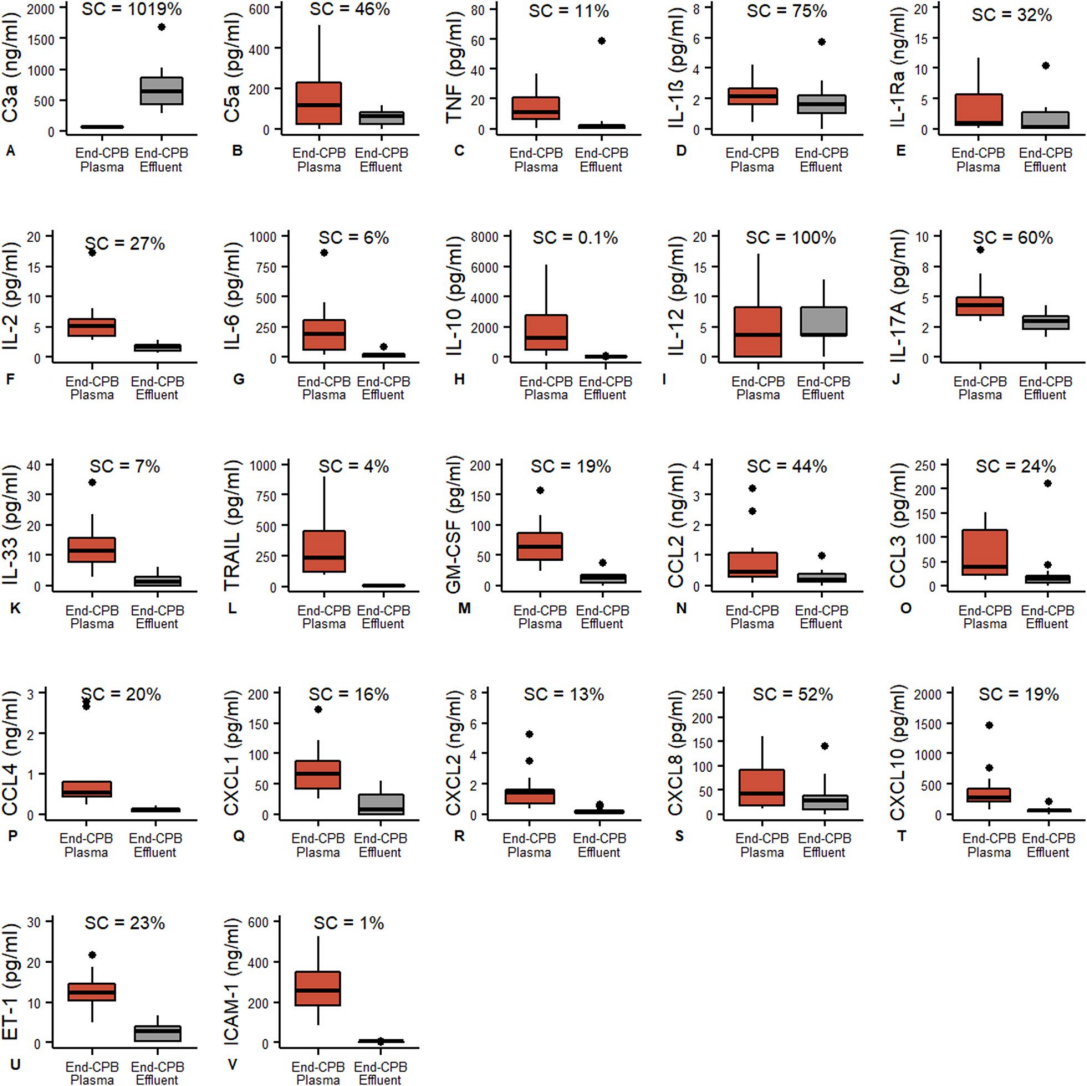
Mediators extracted by SBUF throughout CPB. Sieving coefficient (SC) is calculated by Effluent [mediator]/Plasma [mediator] × 100%

Compared to baseline, circulating plasma concentrations of C2, C3, C3a, C3b, and C5a showed dynamic increases (Fig. 1A–E) during cardiac surgery and CPB, consistent with complement activation, while C1q, C4, C4b, C5, complement factor B/H/I showed no changes at the end of CPB, when compared with baseline values (Fig. 2A–G). Only the relatively low molecular weight anaphylatoxins C3a and C5a were detected in the ultrafiltrate effluent with a sieving coefficient of 1019% and 46%, respectively (Fig. 3A, B). C1q, C2, C3, C4, C5, CFB, CFH and CFI were not detected in the effluent.

### Cytokines and chemokines

**Fig. 4:**
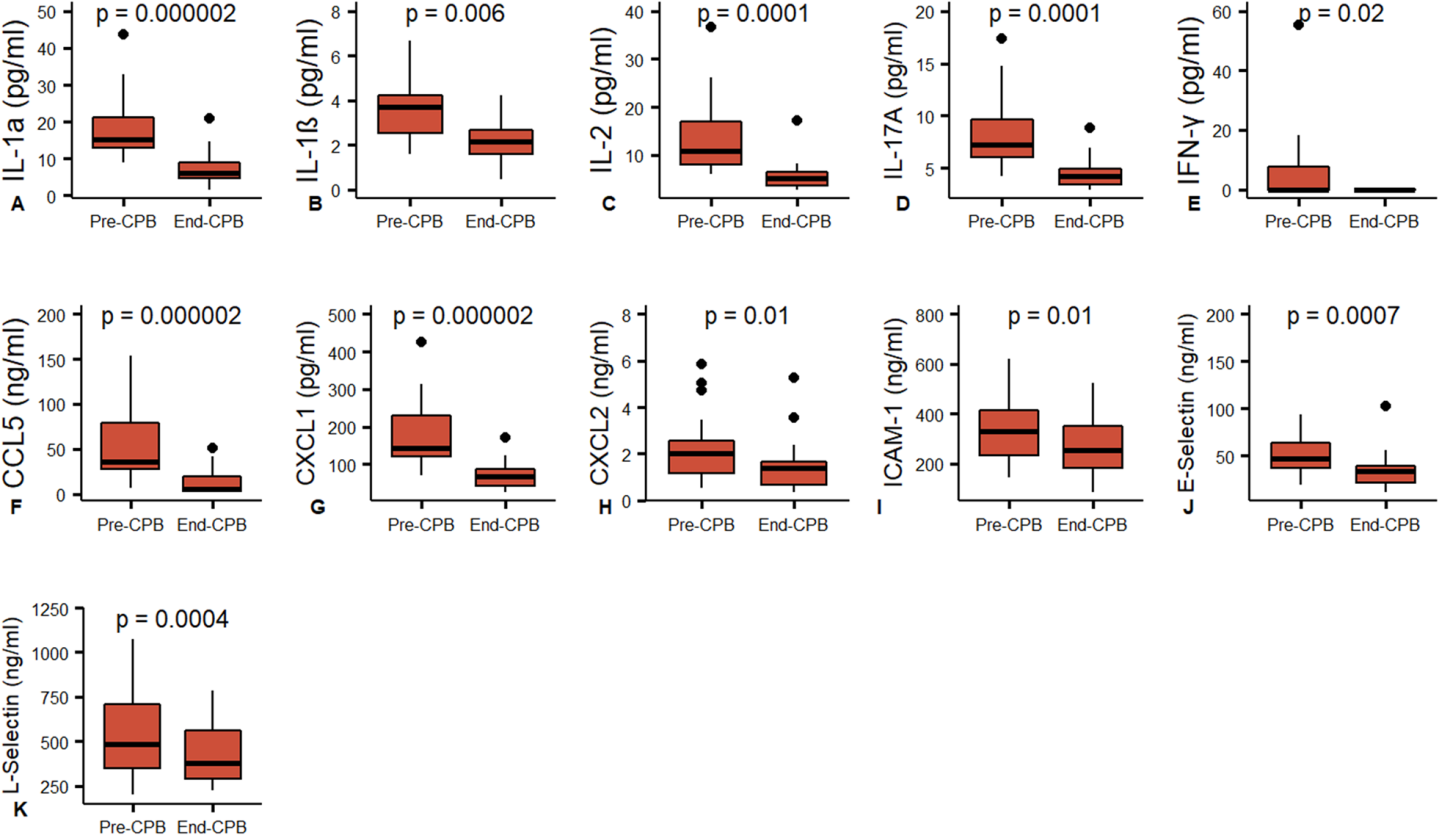
Inflammatory mediators that decrease throughout CPB with SBUF. Paired comparison by Wilcoxon signed-rank test. IL-1β, IL-2, IL-17, CXCL1, CXCL2 and ICAM-1 were extracted by SBUF while IL-1α, IFN-γ, CCL5, L-Selectin and E-Selectin were not

The pro-inflammatory factors IL-6, TRAIL, CCL2, CCL3, CXCL8 (IL-8), CXCL10 and ET-1 showed significant blood concentration increases during the CPB time (Fig. 1G, I–N), while TNF, IL-12, IL-33, GM-CSF and CCL4 showed no changes (Fig. 2H–L). Several factors showed significant decreases in circulating concentrations compared to baseline: IL-1α, IL-1β, IL-2, IL-17A, IFN-γ, CCL5, CXCL1 and CXCL2 (Fig. 4A–H). The anti-inflammatory regulators IL-1Ra and IL-10 also demonstrated reactive changes during CPB (Fig. 1F, H). Most cytokines and chemokines were extracted by ultrafiltration with a range of sieving coefficients from 0.1% to 100% (Fig. 3C–U). Only IL-1α, IFN-γ and CCL5 were not detected in the ultrafiltrate effluent.

### Leukocyte adhesion molecules

Soluble adhesion molecules are frequently elevated in the context of vascular disease or inflammation. Leukocyte adhesion molecules showed static blood concentrations during the CPB with P-selectin and VCAM-1 concentrations not different from baseline (Fig. 2M, N). E-Selectin, L-selectin and ICAM-1 showed slight decreases in plasma over the CPB time (Fig. 4I–K). However, only ICAM-1 was detected in the ultrafiltrate effluent with a sieving coefficient of 1% (Fig. 3V).

### Relationship between molecular mass and extraction

**Fig. 5:**
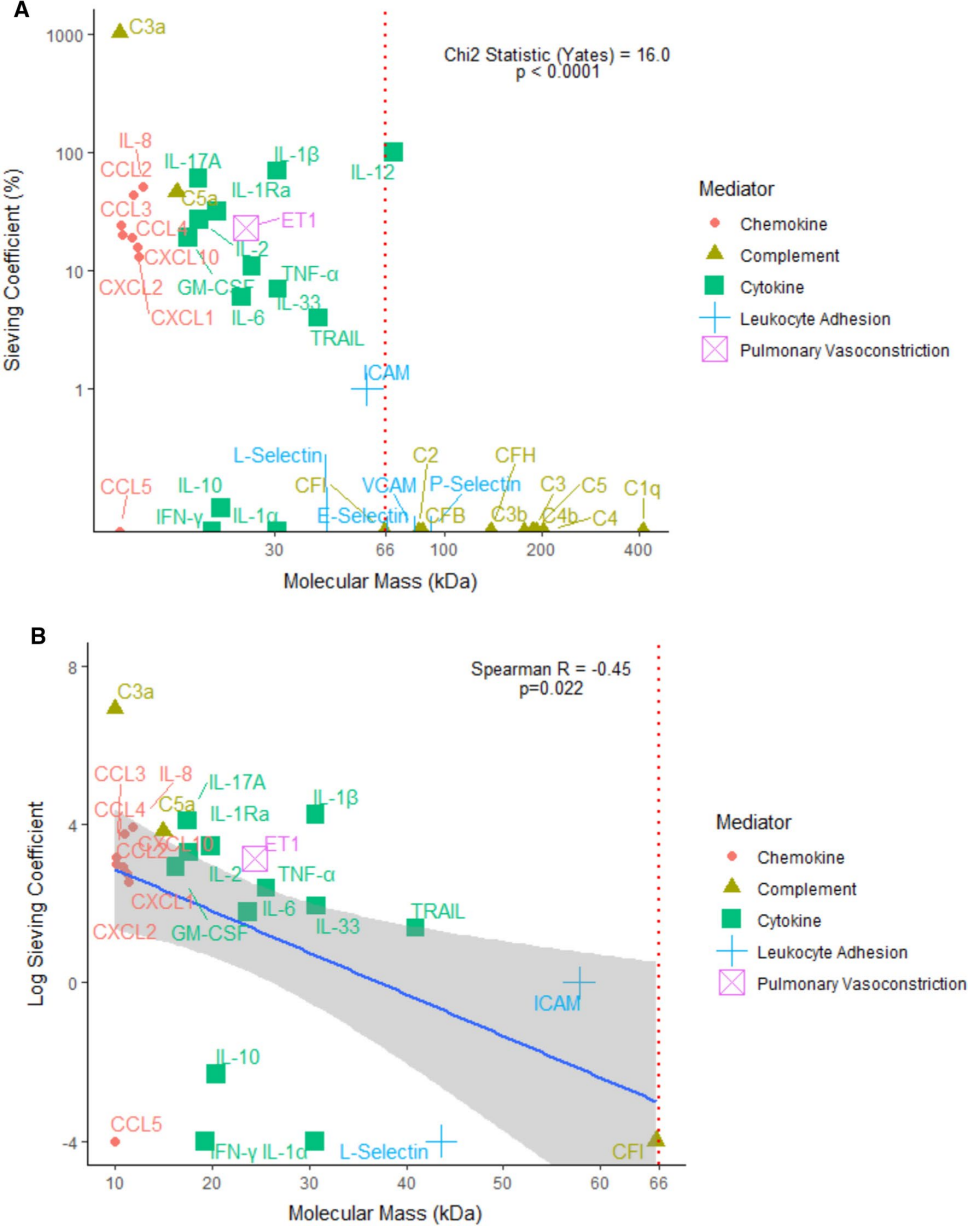
**A** SBUF during CPB extracts several small inflammatory mediators with a range of sieving coefficients. Mediator extraction was associated with molecular mass less than 66 kDa. **B** Mediator molecular mass has a moderate negative correlation with sieving coefficient. Analysis of mediators with molecular mass less than 66 kDa with Spearman’s rank correlation

SBUF is designed to remove lower molecular weight inflammatory mediators preferentially. However, some mediators are tightly associated with other molecules within the plasma and so their removal can be difficult to reliably predict based on molecular weight alone. Mediator extraction by SBUF was, however, statistically associated with a molecular mass of less than 66 kDa (Chi^2^ statistic = 18.8, Yates’ correction = 16.0, p < 0.0001). The relationship between mediator molecular mass and sieving coefficient is displayed in Fig. 5. For those mediators with molecular mass less than 66 kDa, there was a moderate linear association between molecular mass and logarithmic transformation of sieving coefficient with Spearman R = − 0.45 and p = 0.02. This linear relationship was preserved when the five mediators with 0% sieving coefficient were excluded from the analysis (Spearman R = − 0.46 and p = 0.03). Additional file 1: Table S1 shows each mediator’s molecular mass, Pre-CPB plasma concentration, End-CPB plasma concentration, End-CPB effluent concentration and sieving coefficient.

### Impact of glucocorticoids on sieving coefficients

A post-hoc analysis compared 10 patients who received glucocorticoids with prednisone equivalent of 14.5 [11.3–20.9] mg/kg to 10 patients who did not receive any glucocorticoids. Patients who received glucocorticoids were significantly younger (0.2 [0.2–1.6] vs. 12.0 [4.3–30.0] months; p = 0.005), smaller (3.3 [2.6–4.4] vs 6.9 [5.3–13.4] kg; p = 0.009) and had of a higher STAT risk class (4 [4] vs 2[1, 2]; p = 0.001) than patients who did not receive steroids. There was no statistical difference in male sex (80% vs 50%; p = 0.1) or CPB time (270 [140–336] vs. 179 [162–202]; p = 0.39). Of 39 inflammatory mediators measured, only 6 had difference sieving coefficients between the two groups—C5a, TNF, TRAIL, IL-17A, CCL3, CCL4—shown in Table 4.

**Table 4:** Post-Hoc comparison by glucocorticoid (GC) treatment (n = 10 for each group)

	End-CPB [Mediator]			Sieving coefficient		
	GC	No-GC	p-value	GC	No-GC	p-value
C3a (ng/ml)	58.5 [52.8–65.9]	60.1 [41.1–74.9]	1.0	1056% [752%–1596%]	985% [825%–1285%]	0.53
C5a (pg/ml)	241.6 [145.9–303.2]	35.2 [3.3–85.6]	0.005	18% [14%–34%]	180% [47%–400%]	0.03
TNF (pg/ml)	7.4 [6.2–11.0]	18.8 [11.1–32.9]	0.03	26% [19%–39%]	1% [0%–8%]	0.003
TRAIL (pg/ml)	127.2 [106.3–257.8]	288.1 [224.5–456.9]	0.09	7% [6%–8%]	3% [2%–4%]	0.01
IL-17A (pg/ml)	3.6 [2.9–4.3]	4.9 [4.9–6.1]	0.005	83% [69%–117%]	56% [42%–60%]	0.005
CCL3 (pg/ml)	26.4 [20.2–57.2]	77.2 [31.6–346.6]	0.1	55% [27%–72%]	13% [1%–17%]	0.005
CCL4 (ng/ml)	0.5 [0.4–0.6]	0.8 [0.5 –2.2]	0.06	25% [17%–36%]	12% [4%–21%]	0.02

*GC* glucocorticoid-treated group; *No-GC* non-glucocorticoid-treated group

## Discussion

In this study, we evaluated thirty-nine mediators associated with inflammation induction or regulation during pediatric cardiac surgery and CPB with an aim to define those that are dynamically changing and effectively removed by SBUF. Please see Table 5 for a summary of findings table. Ultrafiltration extracted twenty pro-inflammatory factors as well as two regulatory mediators, many of which, are novel to the literature. Activation of the complement pathway is a critical step in promoting inflammation during CBP and was first identified by Kirklin in 1983 [29]. This activation leads to the production of multiple complement products including C3a and C5a, which are anaphylatoxins and stimulate pro-inflammatory immune response through neutrophil chemotaxis and the activation of granulocytes including tissue resident mast cells [4, 30]. The current study supports this paradigm. Interestingly, C3a concentrations double throughout the CPB time but there is an enormous sieving coefficient of 1019% suggesting this anaphylatoxin is a key target of ultrafiltration. C5a had an 18-fold increase between Pre-CPB and End-CPB with a more moderate sieving coefficient of 46%. Andreasson et al*.* identified C3a (sieving coefficient = 122%) and C5a (sieving coefficient = 79%) were removed [15]. Our results showed some differences in the magnitude of mediator extraction as the C3a sieving coefficient was considerably greater for C3a and slightly lower for C5a.

**Table 5:** Summary of findings

	↑ Plasma increase during CPB ↑	~ Plasma no change during CPB ~	↓ Plasma decrease during CPB ↓
Extracted by SBUF	C3a, C5a, IL-1Ra, IL-6, IL-10 CXCL8 (IL-8), CCL2, CCL3, CXCL10, TRAIL, ET1	TNF, IL-12, IL-33, GM-CSF, CCL4	IL-1β, IL-2, IL-17, CXCL1, CXCL2, ICAM-1
Not extracted by SBUF	C2, C3, C3b	C1q, C4, C4b, C5, CFB, CFH, CFI, P-Selectin, VCAM-1	IL-1α, IFN-γ, CCL5, L-Selectin, E-Selectin

Wang et al*.* demonstrated effluent concentrations TNF (7.48 pg/ml), IL-6 (1.2 pg/ml) and IL-8 (0.98 pg/ml) after MUF as compared to TNF (1.5 pg/ml), IL-6 (9.8 pg/ml) and IL-8 (27.8 pg/ml), respectively, reported here after SBUF [18]. Berdat et al*.* also identified TNF, IL-6, and IL-10 in the effluent during non-continuous ultrafiltration [21, 25]. Watanabe et al*.* demonstrated cytokine extraction of IL-6 and IL-8 by ultrafiltration in both children and adults [20]. Therefore, our results further support prior observations that C3a, C5a, TNF, IL-1β, IL-6, IL-8 and IL-10 are extracted by ultrafiltration during pediatric CPB. Given the dynamic temporal responses of inflammatory mediators as well as differences is sample collection timing and analysis procedures, it should be expected that substantial differences in levels of response would be observed between subjects and studies. The IL-8, TNF, IL-1 and the anaphylatoxins have all been associated with neutrophil migration into tissues or subsequent activation therefore a potential clinical benefit from removal of these mediators may be expected.

In addition to confirmatory observations, this was the first investigation to directly identify the extraction of a substantial range of immune regulators and inflammatory mediators in the ultrafiltrate effluent at the end of continuous SBUF. These newly identified extracted mediators included the alarmin IL-33, pro-inflammatory cytokines (IL-2, IL-12, IL-17A, TRAIL, GM-CSF), chemokines associated with monocyte macrophage and T cell recruitment (CCL2, CCL3, CCL4, CXCL1, CXCL2, CXCL10), the leukocyte adhesion molecule ICAM-1, the pulmonary vasoconstrictor ET-1, and anti-inflammatory regulator IL-1Ra. Except for IL-12 (p70), all factors extracted were below the hemoconcentrator membrane’s pore size restriction of 66 kDa. IL-12 (p70) has a size of 70 kDa; however, it is a heterodimer consisting of a p35 (35 kDa) and p40 (40 kDa) subunits that are normally linked by a disulphide bond [26]. Dissociation of IL-12 (p70), degradation to a smaller fragment, or the p35 and p40 subunits associating in the post-membrane effluent could explain these findings.

In contrast, the alarmin IL-1α (30.6 kDa), CCL5 (10.0 kDa) and L-selectin (43.6 kDa) are small molecules but were not detected in the ultrafiltrate effluent. IL-1α End-CPB Plasma concentrations were quite low such that effluent concentrations of IL-1α could easily fall below detectable values (< 10 pg/ml) even if some filtration of this mediator was occurring. CCL5 was well detected in the blood samples at End-CPB but not at all in the effluent. CCL5 is biologically active alone but is known to oligomerize and heterodimerize, precluding ultrafiltration extraction due to molecular weight of CCL5 containing complexes [27]. L-selectin undergoes significant post-translational glycosylation that increases the functional molecular weight to between 65 kDa (expressed in lymphocytes) to 100 kDa (expressed in neutrophils). In its glycosylated states it is, therefore, too large to pass through the membrane [28]. Notably, IFN-γ was not routinely detected in blood samples and not in the effluent at End-CPB, this cytokine would be more commonly observed in situations of ongoing, antigen driven T-cell activation.

Ultrafiltration uses molecular mass, limited by membrane pore size, as the main criteria for extraction. The *Terumo* hemoconcentrator in this study has a cutoff of 66 kDa. The results show that a molecular size above this limit reliably excludes passage across the membrane. This suggests that additional immune modulatory therapies involving the use of monoclonal antibodies or pegylated cytokines would likely remain in circulation despite ultrafiltration. For those mediators with molecular mass less than 66 kDa, a range of sieving coefficients was observed and only a moderate negative linear relationship (Spearman R = − 0.45) between molecular mass and efficiency of extraction. There are likely multiple other properties such as a mediator’s hydrophobicity, molecular structure and quaternary protein interactions that also influence extraction by ultrafiltration.

Consistent with prior investigations, IL-6 and CXCL8 showed dynamic production during CPB and are recognized as early pro-inflammatory signals which recruits neutrophils and whose action may be enhanced by the presence of anaphylatoxins [8, 20, 31]. Interestingly, several major pro-inflammatory factors such as TNF, IL-1α and IL-1β and the chemokines CXCL1, CXLC2 and CC5 showed decreased circulating concentrations by the end of CPB. This observation might be explained by hemodilution upon CPB initiation or extraction of mediators by continuous ultrafiltration without significant production during the CPB time. TNF is known to have a short plasma half-life, especially as a bioactive trimer. It remains likely that monomeric forms of this mediator are selectively depleted during ultrafiltration. Furthermore, IL-1β, CXCL1, CXLC2 and CCL5 are downstream mediators in an inflammatory cascade that would likely be elevated later in the post-operative period.

The anti-inflammatory regulators IL-1Ra and IL-10 are known to be produced early during CPB, which was re-demonstrated in this study [32, 33]. IL-1Ra increased 1.9 × from baseline at the end of CPB and had a sieving coefficient of 33% while IL-10 more dramatically increased 8.7 × with a lower sieving coefficient of 0.1%. IL-10 is a foundational anti-inflammatory mediator which suppresses pro-inflammatory cytokine, leukocyte action and maintains tissue homeostasis [34]. Therefore, the negligible extraction and corresponding accumulation of IL-10 during the CPB time is viewed as a favorable attribute of ultrafiltration. Any depletion of IL-1Ra or IL-10 during ultrafiltration could be deleterious and potentially be modified by supplementation of these mediators, which have been used clinically in multiple trials investigating effects in chronic inflammatory diseases such as Crohn’s disease, ulcerative colitis, rheumatoid arthritis, and systemic lupus erythematosus [34–36].

Glucocorticoids administered prior to CPB, theoretically, could modulate the End-CPB concentrations and ultrafiltration sieving coefficients of NF-κβ mediators. In this study, the patients receiving glucocorticoids were a distinct patient population, as they were largely neonatal patients undergoing complex operations, relative to the non-glucocorticoid group which were older and underwent lower risk procedures. Only 6 of 39 mediators showed statistically different sieving coefficients between the steroid treated and non-treated patients. Generally, the two groups had sieving coefficients within the same order of magnitude and, therefore, glucocorticoid use did not abolish the mediator extraction functionality of ultrafiltration. Interestingly the glucocorticoid group had considerably higher concentrations of C5a, seven times greater, than the non-glucocorticoid group but there was no difference in C3a concentrations. Therefore, steroids do not seem to dampen complement activation during CPB, and the discrepancy of findings between C3a and C5a could be explained by the preferential extraction of C3a (sieving coefficient = 1019%) over C5a (sieving coefficient = 46%) by ultrafiltration. Future well-powered studies are required to primarily assess any interaction between glucocorticoids and ultrafiltration during pediatric cardiac surgery.

The authors recognize limitations in this study. Although all patients underwent cardiac surgery, CPB and continuous ultrafiltration (SBUF), there was a relatively small sample size of 20 patients and significant variation in age, type of congenital heart disease, CPB-time, aortic cross-clamp time, body temperature during CPB and use of DHCA which are all relevant to the regulation inflammatory responses during cardiac surgery and CPB. Second, the *Luminex* immunoassays have limits of analyte detection, which may be limiting to evaluate inflammatory factors or alarmins with low circulating concentrations (e.g. IL-1α). Third, this analysis cannot assess the relationship between the sieving coefficient and the blood flow rate through the hemoconcentrator (this SBUF protocol utilized 5% of calculated cardiac output) or the effluent removal rate (this protocol utilized SBUF effluent rate of 30 ml/kg/hr). Fourth, the exploratory post-hoc analysis assessing the interaction between glucocorticoids and ultrafiltration sieving coefficients should be considered hypothesis-generating only, as it is underpowered and at risk of confounding. Understanding the interplay between these two therapies is essential to understanding the systemic inflammation and anti-inflammatory treatments during CPB and cardiac surgery.

CPB-associated inflammation and post-operative morbidity remains a significant clinical challenge that has yet to be solved. Two recent and well-designed multi-center randomized studies have trialed the use of glucocorticoids (STRESS) and nitric oxide (NITRIC) to treat the adverse immunologic effects of CPB exposure, however, neither were found to improve post-operative clinical outcome for pediatric patients [11, 24]. Therefore, congenital heart disease specialists are keenly interested in solving this issue. Ultrafiltration potentially offers a clinical immunomodulatory effect in addition to a number of other important therapeutic mechanisms—reduction in bleeding complications, prevention of volume overload and organ edema, maintenance of electrolytes and acid–base status—to enhance recovery for infants and children undergoing cardiac surgery [1].

## Conclusion

SBUF during pediatric cardiac surgery and CPB extracts at least twenty-two mediators associated with the regulation of inflammation and early innate immune responses. C3a, C5a, IL-6, IL-8, IL-10, and TNF extraction have been previously identified in this context. We demonstrate for the first time that IL-1β, IL-1Ra, IL-2, IL-12, IL-17A, IL-33, TRAIL, GM-CSF, CCL2, CCL3, CCL4, CXCL1, CXCL2, CXCL10, ICAM-1 and ET1 are also removed by ultrafiltration. Importantly, the key anti-inflammatory mediator IL-10 was not well removed by ultrafiltration and accumulated throughout the CPB time, which is advantageous for returning for immune homeostasis. Ultrafiltration’s extraction of pro-inflammatory mediator adds to other established therapeutic effects including excess volume removal, precise fluid balance control, maintenance of physiology acid–base status and hemoconcentration of coagulation factors to reduce post-operative bleeding complications. Although SBUF clearly extracts many systemic inflammatory factors, translational studies and well-designed randomized trials are required to establish SBUF as an immunomodulatory therapy that enhances recovery after pediatric cardiac surgery with CPB. Furthermore, a broader understanding of the molecular mechanisms of CPB-associated inflammation might identify synergies between other therapies that target key pro-inflammatory mediators, dampen complement activation and propagation or enhance the activity of anti-inflammatory mediators.

## Supplementary Information

**Additional file 1.** Table S1.

## Data Availability

The datasets generated and/or analyzed during the current study are not publicly available due to patient data and information confidentiality but are available from the corresponding author on reasonable request.

## Abbreviations

C: Complement
CF: Complement factor
CCL: CC chemokine ligand
CXCL: CXC chemokine ligand
CPB: Cardiopulmonary bypass
DHCA: Deep hypothermic circulatory arrest
ET1: Endothelin-1
GM-CSF: Granulocyte-macrophage colony-stimulating factor
ICAM: Intracellular adhesion molecule
IL: Interleukin
IFN: Interferon
SC: Sieving coefficient
SBUF: Subzero-balance ultrafiltration
SMUF: Simple modified ultrafiltration
STAT: Society of Thoracic Surgeons-European Association for Cardio-Thoracic Surgery
TCC: Terminal Complement Complex
TNF: Tumor necrosis factor
TRAIL: Tumor necrosis factor-related apoptosis-inducing ligand
VCAM: Vascular cell adhesion molecule
