# Supplementary material for: Novel inflammatory mediator profile observed during pediatric heart surgery with cardiopulmonary bypass and continuous ultrafiltration
Source: J Transl Med. 2023 Jul 5;21:439. doi: 10.1186/s12967-023-04255-8 (PMC10320928; doi:10.1186/s12967-023-04255-8)
Supplement: Supplementary file 1 — Additional file 1. Table S1. [file 12967_2023_4255_MOESM1_ESM.docx]

**Additional file 1**

| **Table S1** | | | | | |
| --- | --- | --- | --- | --- | --- |
| **Mediator** | **Molecular Mass (kDa)** | **Pre-CPB**  **Plasma** | **End-CPB**  **Plasma** | **End-CPB**  **Effluent** | **Sieving Coefficient** |
| **C1q** (µg/ml) | 410.0 | 27.7 [18.9-35.7] | 31.7 [22.9-43.3] | 0 [0-0] | 0% [0%-0%] |
| **C2** (ng/ml) | 83.3 | 210.9 [55.3-302.9] | 740.0 [501.9-1099] | 0 [0-0] | 0% [0%-0%] |
| **C3** (µg/ml) | 187.1 | 7.7 [4.5-10.2] | 54.1 [19.6-66.9] | 0 [0-0] | 0% [0%-0%] |
| **C3a** (ng/ml) | 10.0 | 23.9 [13.9-28.6] | 58.5 [52.2-68.8] | 639.0 [425.5-868.6] | 1019% [788%-1348%] |
| **C3b** (µg/ml) | 177.0 | 1.3 [0-13.5] | 294.4 [160.5-389.4] | 0 [0-0] | 0% [0%-0%] |
| **C4** (µg/ml) | 203.0 | 82.0 [63.2-98.1] | 95.6 [76.4-120.0] | 0 [0-0] | 0% [0%-0%] |
| **C4b** (µg/ml) | 192.7 | 5.8 [4.7-6.5] | 5.8 [4.2-7.1] | 0 [0-0] | 0% [0%-0%] |
| **C5** (µg/ml) | 188.3 | 13.9 [5.4-17.3] | 10.1 [7.1-15.0] | 0 [0-0] | 0% [0%-0%] |
| **C5a** (pg/ml) | 15.0 | 6.5 [0-99.5] | 117.7 [22.4-228.2] | 61.3 [22.5-80.8] | 46% [17%-198] |
| **CFB** (µg/ml) | 85.5 | 78.7 [61.4-96.8] | 91.7 [72.7-112.1] | 0 [0-0] | 0% [0%-0%] |
| **CFH** (µg/ml) | 139.1 | 95.5 [73.5-113.5] | 123.5 [92.2-168.3] | 0 [0-0] | 0% [0%-0%] |
| **CFI** (µg/ml) | 65.7 | 11.1 [7.4-13.7] | 12.4 [8.6-14.7] | 0 [0-0] | 0% [0%-0%] |
| **ET-1** (pg/ml) | 24.4 | 10.0 [7.5-13.1] | 12.5 [10.4-14.6] | 2.6 [0.2-4.1] | 22% [2%-36%] |
| **TNF** (pg/ml) | 25.6 | 20.7 [17.6-24.1] | 11.1 [6.2-21.3] | 1.5 [0.2-2.7] | 11% [0%-25%] |
| **IL-1α** (pg/ml) | 30.6 | 15.3 [12.9-21.2] | 6.1 [4.5-8.9] | 0 [0-0] | 0% [0%-0%] |
| **IL-1β** (pg/ml) | 30.7 | 3.7 [2.5-4.2] | 2.1 [1.6-2.7] | 1.6 [1.0-2.2] | 77% [46%-100%] |
| **IL-1Ra** (ng/ml) | 19.9 | 0.3 [0.3-0.9] | 0.9 [0.6-5.7] | 0.2 [0.1-2.7] | 33% [26%-53%] |
| **IL-2** (pg/ml) | 17.6 | 10.8 [8.0-16.9] | 5.1 [3.5-6.3] | 1.7 [1.0-2.1] | 27% [21%-43%] |
| **IL-6** (pg/ml) | 23.7 | 20.0 [12.4-36.6] | 191.4 [58.3-303.9] | 9.8 [2.8-31.0] | 7% [2%-10%] |
| **IL-10** (pg/ml) | 20.5 | 174.7 [108.3-262.1] | 1266.7 [508.7-2795.7] | 3.2 [0-17.6] | 0.1% [0.1%-2.0%] |
| **IL-12** (pg/ml) | 70.0 | 8.3 [3.6-8.3] | 3.6 [0-8.3] | 3.6 [3.6-8.3] | 100% [47%-INF] |
| **IL-17A** (pg/ml) | 17.5 | 7.2 [6.1-9.7] | 4.3 [3.4-4.9] | 2.9 [2.3-3.4] | 60% [49%-82%] |
| **IL-33** (pg/ml) | 30.8 | 11.0 [7.9-16.2] | 11.4 [7.9-15.8] | 1.2 [0-2.9] | 7% [0%-18%] |
| **TRAIL** (pg/ml) | 41.0 | 113.2 [98.6-165.4] | 234.0 [120.2-452.9] | 9.3 [9.3-15.0] | 4% [3%-8%] |
| **IFN-γ** (pg/ml) | 19.3 | 0 [0-7.5] | 0 [0-0] | 0 [0-0] | NA |
| **GM-CSF** (pg/ml) | 16.3 | 58.9 [32.4-72.7] | 63.8 [42.1-86.7] | 12.4 [4.9-17.9] | 19% [11%-26%] |
| **CCL2** (ng/ml) | 11.0 | 0.2 [0.2-0.3] | 0.5 [0.3-1.1] | 0.2 [0.1-0.4] | 44% [31%-55%] |
| **CCL3** (pg/ml) | 10.1 | 28.3 [23.3-32.8] | 39.3 [22.3-114.6] | 14.4 [7.1-19.5] | 24% [10%-56%] |
| **CCL4** (ng/ml) | 10.2 | 0.5 [0.4-0.6] | 0.5 [0.4-0.8] | 0.1 [0.1-0.1] | 20% [7%-26%] |
| **CCL5** (ng/ml) | 10.0 | 36.0 [27.5-78.7] | 5.2 [2.5-19.2] | 0 [0-0] | 0% [0%-0%] |
| **CXCL1** (pg/ml) | 11.3 | 142.0 [121.9-229.5] | 66.5 [42.7-87.5] | 7.7 [0-32.9] | 16% [0%-38%] |
| **CXCL2** (ng/ml) | 11.4 | 2.0 [1.2-2.6] | 1.4 [0.7-1.6] | 0.1 [0.1-0.2] | 13% [7%-20%] |
| **CXCL8** (pg/ml) | 11.8 | 12.6 [9.1-24.2] | 41.9 [17.0-92.3] | 27.8 [9.7-39.1] | 52% [39%-66%] |
| **CXCL10** (pg/ml) | 10.9 | 203.6 [117.21-298.1] | 267.8 [200.2-421.35] | 56.7 [35.8-78.33] | 19% [16%-23%] |
| **E-Selectin** (ng/ml) | 66.7 | 47.3 [37.5-63.5] | 33.3 [21.7-39.1] | 0 [0-0] | 0% [0%-0%] |
| **L-Selectin** (ng/ml) | 43.6 | 485.2 [348.0-707.1] | 377.4 [287.9-562.0] | 0 [0-0] | 0% [0%-0%] |
| **P-Selectin** (ng/ml) | 90.8 | 32.9 [27.9-38.8] | 32.9 [27.3-37.1] | 0 [0-0] | 0% [0%-0%] |
| **ICAM-1** (ng/ml) | 57.8 | 329.7 [233.5-411.7] | 255.2 [181.7-350.8] | 4.1 [2.8-4.5] | 1% [0%-2%] |
| **VCAM-1** (µg/ml) | 81.3 | 1.0 [0.6-1.6] | 0.8 [0.6-1.4] | 0 [0-0] | 0% [0%-0%] |
| Presented as median [interquartile range] | | | | | |
